# Building blocks of life: improving nutrition and health outcomes through fortification and breastfeeding in the first 1000 days of a child’s life

**DOI:** 10.3389/fnut.2025.1517247

**Published:** 2025-03-03

**Authors:** Rohini Saran, Jayendra Kasar, Meenakshi Jha, Jatindra K. Sahu, Sonu S. Babu, Ankur Mutreja

**Affiliations:** ^1^PATH, New Delhi, India; ^2^National Institute of Public Cooperation and Child Development, New Delhi, India; ^3^Centre for Rural Development and Technology, Indian Institute of Technology, New Delhi, India

**Keywords:** food fortification, first 1000 days, rice fortification, breastfeeding, human milk banking, early childhood development, nutrition, global south

## Abstract

Micronutrient deficiencies continue to be an important concern in India, particularly among mothers and young children. Food fortification and fortified rice as a scalable and cost-effective solution, has been accepted as a critical intervention to address these deficiencies. Our analysis examines how food fortification can improve the health of mothers and children in India. Focusing on the vital window of first 1,000 days since conception of a child, we examine the benefits of food fortification, the incidence of micronutrient deficiencies, and the incorporation of fortified foods into India’s social safety net program. The analysis emphasizes the importance of successful public-private partnerships, local institutional commitment, and long-term political commitment to the success of fortification programs. We further emphasize that to ensure optimal nutrition during this critical stage, it essential to support breastfeeding, establish human milk banks, and encourage complementary feeding alternatives. Policymakers, program implementers, and stakeholders committed to improving maternal and child health outcomes in India will benefit from our findings.

## 1 Introduction

The first 1,000 days, from conception to a child’s second birthday, represent a crucial window for impactful interventions in health and nutrition. During this period, children are most vulnerable and in critical need of optimal nutrition to support healthy growth, brain development, and long-term health (1). This stage lays the groundwork for the foundations of a child’s physical, cognitive, and emotional development, with nutrition playing a vital role in these outcomes (1). By the age of 2 years, 80% of a child’s brain is developed. Optimal nutrition of mother during pregnancy, post-partum period and of a child during early years of life plays a critical role to develop, learn and thrive (1).

**Figure 1:**
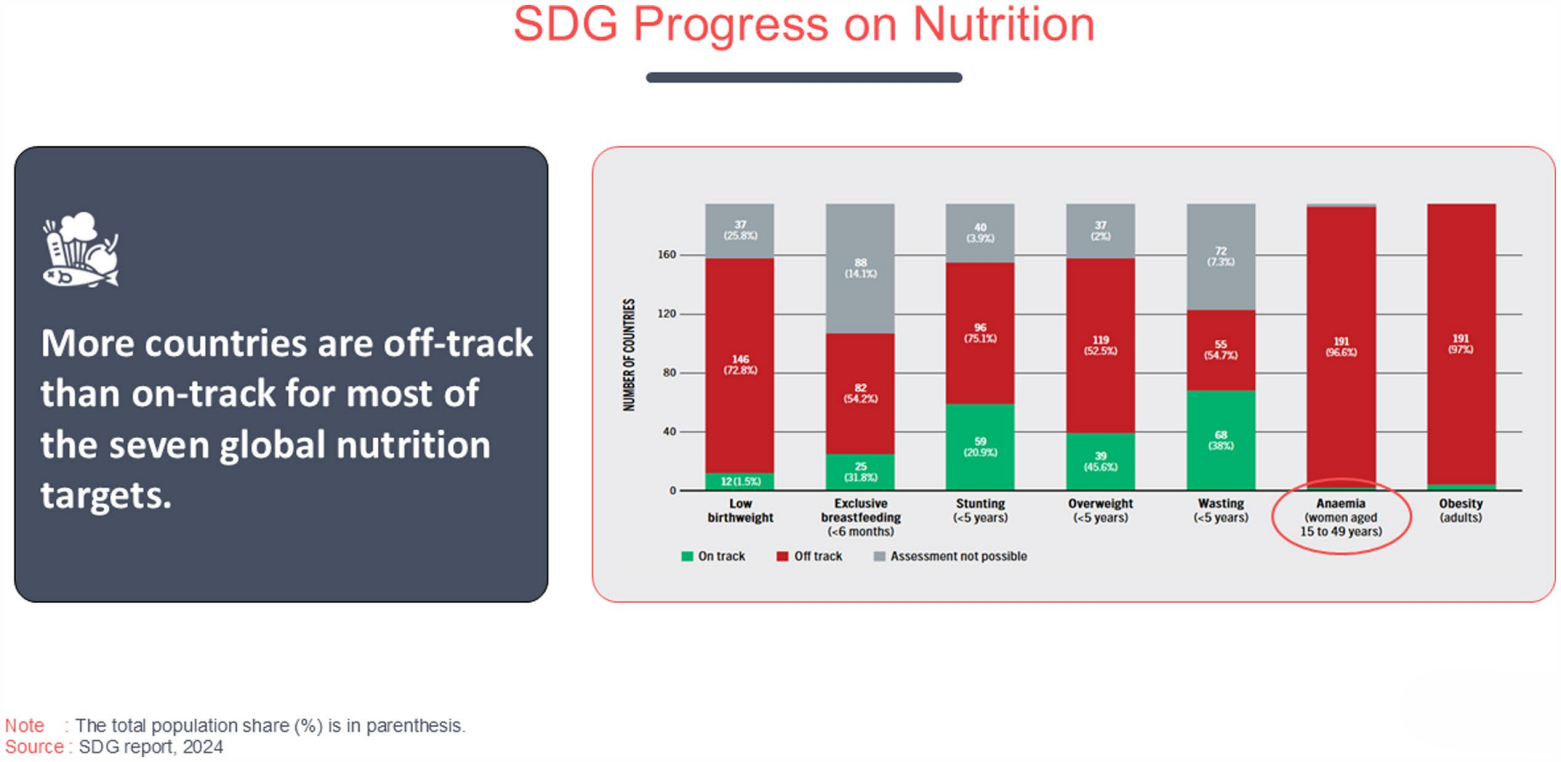
SDG progress on nutrition.

**Figure 2:**
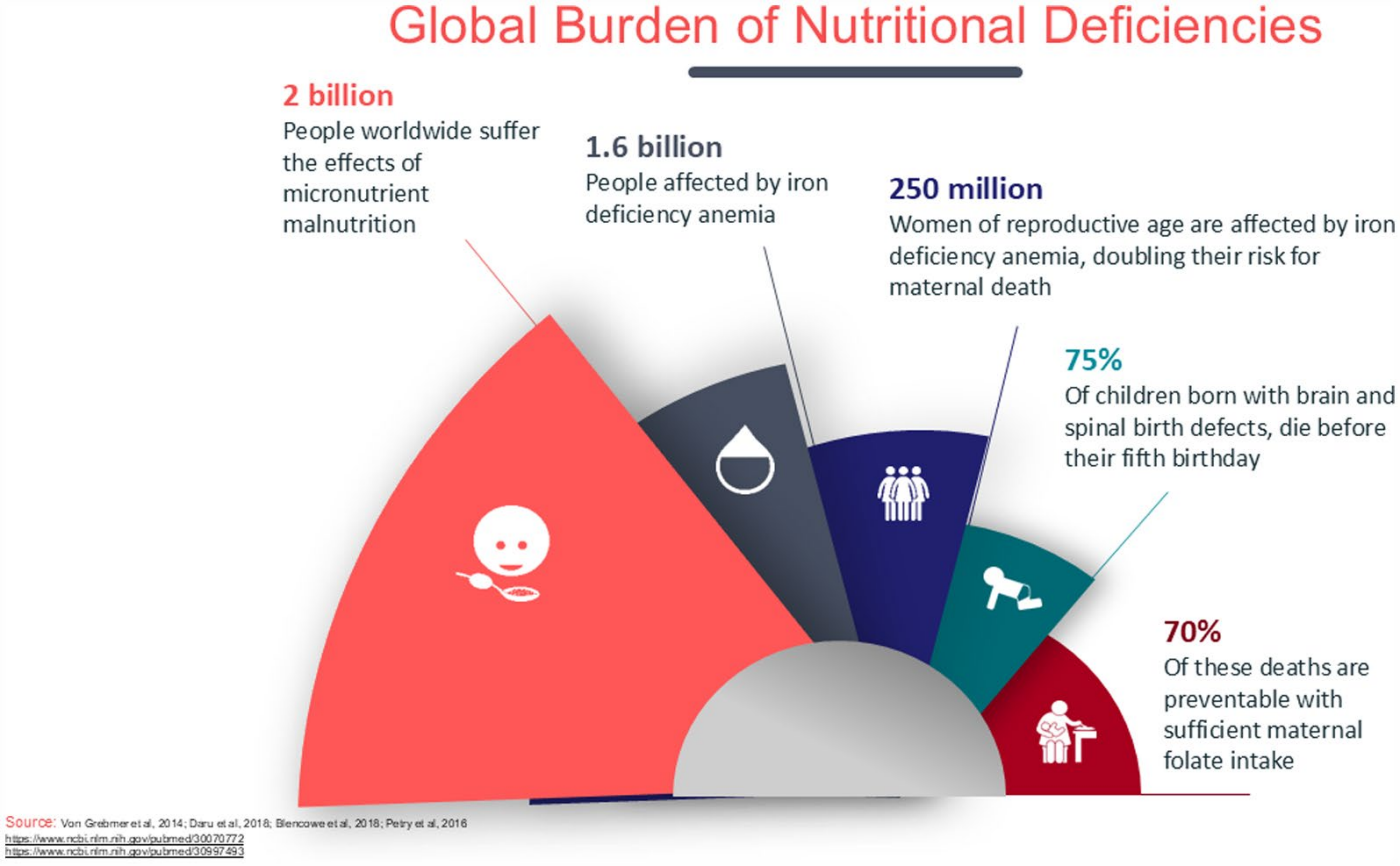
Global burden of nutritional deficiencies.

The SDG report 2024 (2) emphasizes that more countries are off-track than on-track for the seven global nutrition targets. Key indicators such as low birthweight, exclusive breastfeeding, stunting, overweight in children under 5 years, wasting, anemia in women aged 15–49, and obesity show significant challenges. The chart reveals the stark reality that a majority of countries fall into the “off-track” category, particularly for both anemia and obesity (191 countries). A small number of countries have managed to achieve “on-track” status for targets like exclusive breastfeeding and low birthweight, but these remain exceptions rather than the norm. Hunger continues to pose a significant challenge globally, with Africa having the highest percentage of its population affected at 20.4%, which translates to 298.4 million people in 2023. Although the prevalence is lower in Asia at 8.1%, it accounts for 384.5 million people, making it the region with the largest absolute number of individuals facing hunger—more than half of the global total. Latin America and the Caribbean face hunger rates of 6.2%, affecting 41 million people, while Oceania, with a hunger rate of 7.3%, has 3.3 million people affected (3). The inability to assess progress in some areas further underscores the complexities in addressing global nutrition challenges (Figure 1) usually characterized by persistent undernutrition and micronutrient deficiencies that impact the population’s overall health and development (4). Despite several strategies that have been proposed to provide target nutrients to infants and young children, optimal nutrition during the first 1,000 days since conception remains a significant public health challenge (Figure 2). The strategies include diversified diets, fortified complementary foods, fortified animal milk, micronutrient powders, and small quantities of lipid-based nutrient supplements (4).

During the first 1,000 days of life, eight key nutrients namely carotenoids (lutein and zeaxanthin), choline, folate, iodine, iron, omega-3 fatty acids, and vitamin D play essential roles. Other important nutrients for maternal, newborn, and toddler health include magnesium, vitamin A, B vitamins, and additional trace minerals. Iron fortification led to a significant increase in serum ferritin and hemoglobin levels in women of reproductive age and pregnant women. Folate fortification significantly reduced the incidence of congenital abnormalities like neural tube defects without increasing the incidence of twinning (5).

In 2020, approximately 149 million children under the age of five worldwide were impacted by stunting, and 49 million suffered from wasting (6, 7). The prevalence of maternal and child undernutrition in low-income and middle-income countries (LMICs) has remained unacceptably high (8). Stunting often begins in utero and increases, on average, for at least the first 2 years after birth (9). Evidence indicates that stunting becomes largely irreversible after the first 1,000 days of a child’s life, perpetuating an intergenerational cycle of impaired growth and development (10). Folic acid and iodine are particularly critical for fetal neurodevelopment but are often lacking in the diets of pregnant women (11). India has made substantial strides in reducing child mortality and improving child survival (12). The Government of India has implemented various programs that target childhood illnesses, lower mortality rates, and promote early childhood development (13).

**Figure 3:**
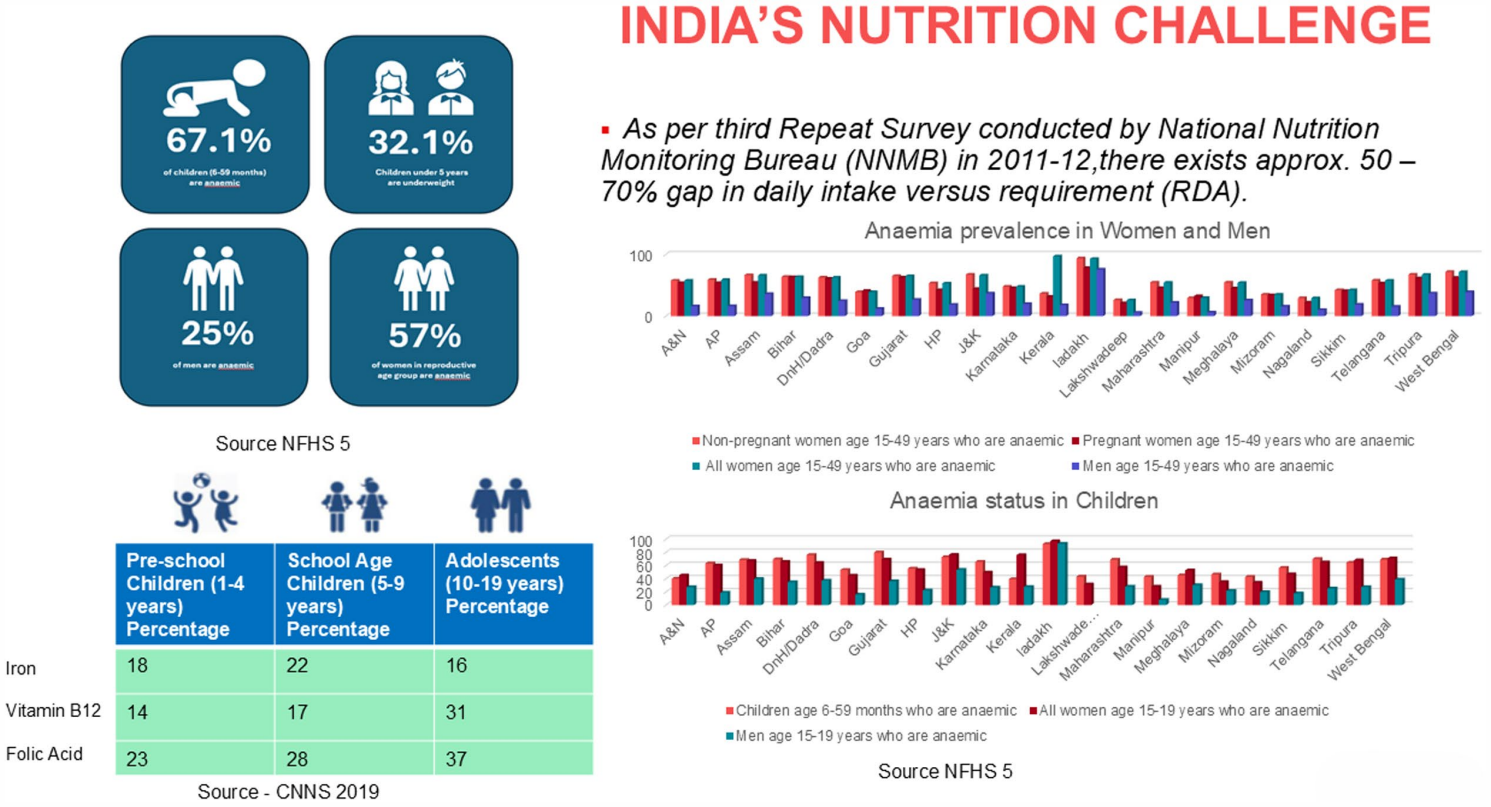
India’s nutrition challenge.

The latest National Family Health Survey (NFHS-5) (2019–21) (14) demonstrates some progress, with stunting reduced by 2.9%, wasting by 1.7%, and underweight prevalence by 3.7% compared to NFHS-4 (2015–16) (15). However, the prevalence of anemia remains high (Figure 3) (14), with over half of Indian women still affected, signaling an ongoing need for targeted nutritional interventions. Addressing this triple burden of malnutrition—including undernutrition, micronutrient deficiencies, and rising levels of obesity in children—requires a timely, comprehensive, and scalable approach to ensure adequate nutrition during the first 1,000 days. The quantity of food consumed by an infant is limited, and hence it is imperative to provide optimum nutrition in every meal. Home-based meals may not be adequate, and can leave gaps especially when the diet is predominantly vegetarian or if there is increased consumption of nutrients that adversely affect iron absorption (16, 17).

The initial 1,000 days cover the period from pregnancy through a child’s second year, requiring an adequate supply of macro-and micronutrients for mothers and infants to promote proper fetal and early childhood development (9). Adequate caloric intake and nutrients such as iron, zinc, folate, iodine, and vitamin D are critical in preventing malnutrition-related complications such as low birth weight, anemia, and impaired cognitive development (9). Nutritional supplementation during the first 1,000 days of life have shown improvements in birth and growth outcomes and newborn stunting (9). Compared to supplementation with iron alone or with iron and folic acid, multiple-micronutrient supplementation that includes iron and folic acid lowers the risk of small-for-gestational-age births, low birth weight, and stillbirth (18).

Multiple interventions contribute significantly to child development, including ensuring optimal maternal nutrition during pregnancy, iron and folic acid supplementation, early initiation of breastfeeding, and exclusive breastfeeding for the first 6 months of life (18). For small and sick newborns, access to donor human milk when needed and fortification of human milk as per clinical recommendations are crucial (19). When Mother’s Own Milk (MOM) is unavailable or insufficient, donor human milk (DHM) is considered the optimal alternative, as recommended by the American Academy of Pediatrics, the European Society for Paediatric Gastroenterology, Hepatology and Nutrition, and the World Health Organization (20). Introducing nutritionally adequate and safe complementary (solid) foods at 6 months, alongside continued breastfeeding up to 2 years or beyond, further supports growth and development (18). Additionally, food fortification and addressing broader social determinants play essential roles in fostering child health (21, 22).

Fortifying staple foods with vital vitamins and minerals is an effective strategy for combating common nutritional deficiencies, ensuring that pregnant women and infants receive the essential nutrients needed for optimal development (18). This proactive approach significantly reduces the risk of malnutrition-related issues, supports healthy fetal growth, and promotes overall childhood development, ultimately leading to better health outcomes later in life.

In this review, we will explore interventions focused on achieving optimal nutrition during the crucial first 1,000 days of life, with a particular emphasis on food fortification.

## 2 Methodology

To explore the role of food fortification as a public health strategy to address nutritional challenges during the first 1,000 days of a child’s life we used a structured methodology of focused literature search conducted across databases such as PubMed, Google Scholar, Scopus and organizational repositories, including WHO, UNICEF, and FAO. Keywords such as “food fortification,” “maternal nutrition,” “child nutrition,” “nutritional challenges,” “policy review,” “breast feeding,” “donor human milk” and “first 1,000 days” were used in various combinations to refine the search results. Studies related to both global and Indian fortification programs, policy notes, and opinion articles were included in addition to randomized controlled trials (RCTs), cohort studies, systematic reviews, case–control studies, and programmatic evaluations that addressed food fortification interventions and their impact on maternal and child health outcomes. Studies and guidelines related to human milk banks and programmatic experiences were also included to provide a comprehensive perspective. Studies that were not relevant to the target population group (mothers and children) or government programs were excluded along with studies focused on disease populations or conditions unrelated to the core topic of food fortification and maternal and child health.

The inclusion process also involved an initial screening of titles and abstracts to identify relevant sources, followed by a detailed review of full-text articles to extract information on strategies, implementation, outcomes, and challenges. Reference lists from key articles were cross-referenced to identify additional relevant materials. Expert consultations complemented the literature review, offering practical insights into the implementation, challenges, and policy implications of food fortification interventions. These consultations captured expert opinions of program implementers, public health professionals, and policymakers engaged in maternal and child health programs. The findings from the literature and expert insights were co-analyzed to identify gaps, highlight successful practices, and provide recommendations for strengthening food fortification policies and programs.

## 3 Results

### 3.1 Food fortification as a nutritional intervention

Food fortification involves the deliberate addition of essential vitamins and minerals to widely consumed foods to combat nutritional deficiencies (23). This intervention is both cost-effective and scalable (23), making it particularly relevant for public health improvement in low-resource settings where micronutrient deficiencies are prevalent (23). The practice of food fortification has a rich history, with early initiatives such as the iodization of salt in the 1920s aimed at reducing the incidence of goiter (23). The primary rationale behind fortification is to increase the intake of micronutrients in populations where dietary deficiencies are widespread (24–26), without necessitating significant alterations to individual food consumption patterns. Fortification is especially beneficial when applied to staple foods, as it guarantees that a large segment of the population receives the intended health benefits. A review of micronutrient fortification studies highlights notable improvements in nutrient levels and health indicators (5). Iron fortification, studied in 13 trials, increased hemoglobin concentration (Standardized Mean Difference SMD: 0.64, 95% Confidence Interval CI: 0.30–0.97), serum ferritin (SMD: 0.41, 95% CI: 0.23–0.60), and reduced anemia prevalence (Relative Risk RR: 0.68, 95% CI: 0.49–0.93), particularly benefiting iron-deficient populations (5). Zinc fortification (10 studies) improved serum zinc levels (SMD: 1.28, 95% CI: 1.06–1.51), while vitamin D fortification (13 RCTs) raised serum vitamin D (SMD: 1.23, 95% CI: 1.03–1.43) and decreased parathyroid hormone (SMD: –0.40, 95% CI: −0.78 to −0.02) (5). Vitamin A fortification improved serum retinol (SMD: 0.61, 95% CI: 0.34–0.89) and hemoglobin (SMD: 0.48, 95% CI: 0.17–0.80), while iodine fortification (16 studies) showed a significant rise in urinary iodine levels (SMD: 6.39, 95% CI: 4.85–7.93) and a reduction in hypothyroidism (RR: 1.42, 95% CI: 1.11–1.82). Dual iron and iodine fortification improved hemoglobin (SMD: 0.53, 95% CI: 0.16–0.89) and reduced anemia by 53% (RR: 0.47, 95% CI: 0.29–0.78). Multiple micronutrient (MMN) fortification increased hemoglobin (SMD: 0.75, 95% CI: 0.38–1.13) and reduced anemia (RR: 0.55, 95% CI: 0.42–0.72). Folate fortification, examined across 31 studies, notably reduced neural tube defects by 43% (RR: 0.57, 95% CI: 0.45–0.73) (5). Fortification is especially beneficial when applied to staple foods, as it guarantees that a large segment of the population receives the intended health benefits (27). The selection of natural fortificants in the fortification method depends upon various factors, like, (a) diverse micronutrients available in food, (b) quantity of micronutrients present in food, (c) types of micronutrient-rich foods, (d) influence of food vehicle on sensory characteristics and (e) consumer acceptability (27). Additionally, micronutrient fortification considers food preferences based on the dimensions of a culturally sustainable diet. Thus, we re-emphasize that investing in micronutrient fortification, which is also environmentally, culturally and economically sustainable, could play a significant role in preventing and controlling micronutrient deficiencies, improving diets (28).

A systematic review and meta-analysis encompassing 50 studies has addressed a crucial knowledge gap regarding the comprehensive impact of large-scale fortification programs in low-and middle-income countries (LMIC) (26). These programs, which focus on micronutrients such as iodine, folic acid, vitamin A, and iron, have resulted in significant health improvements in LMICs, as exemplified by:

- A 34% reduction in anemia attributable to improved iron stores, with findings indicating that all age groups benefited, particularly vulnerable populations like women of reproductive age and pregnant women (29).
- A 41% decrease in the odds of neural tube defects due to reduced folate deficiency among women of reproductive age (29).
- A notable reduction in vitamin A deficiency (VAD), positively impacting approximately 3 million children (aged 0–9 years) within a single year and significantly lowering their mortality risk (29).
- A World Health Organization (WHO) meta-analysis revealing that rice fortified with vitamins and minerals, including iron, enhances iron status by potentially reducing the risk of iron deficiency by 35% and increasing average hemoglobin concentration by nearly 2 g/L (30). Measurable improvements in the micronutrient and health status of women and children are possible with large scale food fortification (LSFF) (29).

Despite adequate food production, India’s per capita nutrition intake ranks among the lowest globally. Although the per capita availability of various foods aligns with the Recommended Dietary Intake (RDI), the distribution of these foods is disproportionately unfavorable to vulnerable groups due to limited income and purchasing power. The ordinary Indian population predominantly relies on plant-based diets, which often lack essential nutrients. Micronutrient deficiency in the Indian diet poses a significant health challenge, impacting millions across the nation. The inadequate intake of essential vitamins and minerals, such as iron, zinc, vitamin A, and iodine, can lead to severe health issues, including anaemia, impaired cognitive development, and compromised immune function (31). Plant based diet might negatively impact iron absorption due to the presence of certain natural inhibitors (32). Plant foods contain compounds such as phytates (found in grains and legumes) and tannins (in tea and coffee), which can reduce the body’s ability to absorb non-heme iron. Additionally, low bioavailability of iron from plant sources poses challenges, as the absorption rate for non-heme iron is significantly lower. Furthermore, uncalibrated vegetarian and vegan diets may also lack key nutrients like vitamin C, which helps enhance iron absorption, further compounding the issue (32, 33). Micronutrient fortification of staple foods adds essential vitamins and minerals without altering the taste, smell, or appearance of the food, ensuring no organoleptic changes. This makes it an effective and sustainable solution to prevent nutrient deficiencies across populations without requiring considerable behavioral changes. It is cost-effective and can significantly improve public health by proactively addressing micronutrient gaps. The fortification process typically entails the addition of minute quantities of nutrients—such as vitamin A, iron, and iodine—to staple foods like rice, wheat flour, oil, and milk (34). Therefore, increasing consumption of a variety of plant-based foods, in combination with food fortification and supplementation where needed, is recommended for children and adolescents to have sustainable and nutritionally adequate diets (35).

Globally, food fortification programs have been implemented with great success. For example, in the United States, the fortification of flour with folic acid has significantly reduced the incidence of neural tube defects (36). In South Africa, the fortification of maize and wheat with vitamins A and D has contributed to the reduction of micronutrient deficiencies (37, 38). These case studies highlight the effectiveness of fortification in improving public health outcomes (39). India has managed to scale the rice fortification program to feed nearly 800 million people (40). Since rice is a staple diet in many Indian states, fortifying rice is seen as one of the most affordable and sustainable ways to enhance health outcomes. Research has demonstrated that adding fortified rice to the diet is effective in improving hemoglobin levels, thereby reducing the prevalence of anemia and enhancing cognitive performance (41). However, despite its success, in places, large scale food fortification (LSFF) faces several challenges. Certain foods can be particularly challenging to fortify due to their unique characteristics, how they are processed, and the way people expect them to look, taste, and feel. For example, adding nutrients to beverages like soft drinks or juices can easily affect their taste or cause unwanted sediment, making them less appealing (42). Rice, which is a staple food in many parts of the world, is difficult to fortify consistently, especially in areas where small-scale milling is common. Dairy products, like milk, often face issues with sensory changes or nutrient stability, as added nutrients can interact with milk’s natural components and reduce their effectiveness (42). Cereal-based foods, such as snacks or breakfast cereals, risk changes in texture or flavor when fortified, while condiments like soy sauce or fish sauce require careful attention to avoid altering their distinct and familiar tastes (42). Similarly, baked goods present a unique challenge because the high baking temperatures can break down heat-sensitive vitamins like thiamine (42).

### 3.2 Donor human Milk and fortification for vulnerable babies

Feeding premature, LBW, and small-for-gestational-age babies comes with additional challenges, such as prolonged separation of mothers and babies, delay in attachment to breast, low priority given to breastfeeding by physicians, and lack of skilled counselling and hands on support to mothers to breastfeed and express milk (54, 55). Moreover, a large number of babies do not receive mother’s milk due to reasons, such as mother’s illness, death, abandonment, delay in milk production, leaving them vulnerable, especially if they are born premature. Technological advances in human milk banking have revolutionized the way donor milk is collected, processed, stored, and distributed, ensuring higher safety and quality standards. Innovations such as automated pasteurization systems and real-time milk testing devices have significantly improved the efficiency and accuracy of pathogen elimination while preserving the essential nutrients and bioactive components of human milk (56).

As per a landscape study conducted by PATH and Lokmanya Tilak Municipal Medical College and General Hospital (LTMMC and GH), 30 to 50 percent of vulnerable babies admitted in the neonatal intensive care units (NICU) and 10 to 20 percent of term (healthy) babies in India do not have access to mother’s own milk (MOM) to meet their short-or long-term needs (57). Alternatively, these babies are given formula milk or cow’s milk. For such vulnerable infants, donor human milk is the next best option when a mother’s own milk is unavailable, offering critical support in reducing the risk of infections, necrotizing enterocolitis, and other complications in these vulnerable infants. By ensuring access to safe, pasteurized human milk, milk banks play a crucial role in bridging nutritional gaps, promoting healthy development, and improving survival rates in this high-risk population, laying the foundation for a healthier start in life, especially newborns admitted in sick newborn care units (SNCU) or NICU.

Also to prevent extrauterine growth restriction (EUGR), which is linked to poor neurocognitive outcomes and specific nutrient deficiencies, nutrient fortification of human milk (HM) is recommended for very low birth weight (VLBW) infants (58). Current fortification methods include: 1. Standard fortification, and 2. Individualized fortification, which encompasses “Adjustable fortification” and “Targeted fortification” (59). Despite standard fortification, many very VLBW infants still experience suboptimal growth, highlighting the need for optimized human milk fortification. Adjustable fortification has been shown to enhance protein intake, support somatic and head growth, and appears to be a practical method for optimizing HM fortification. While targeted fortification has demonstrated feasibility and effectiveness in some trials, further improvements are needed. Additionally, enhancing the quality of human milk fortifiers (HMF) is crucial. Although HM-based fortifiers show promise, with some studies indicating benefits in morbidity and mortality for infants fed an exclusively human milk-based diet, concerns remain regarding their efficacy, safety, and ethical implications (19, 59).

**Figure 6:**
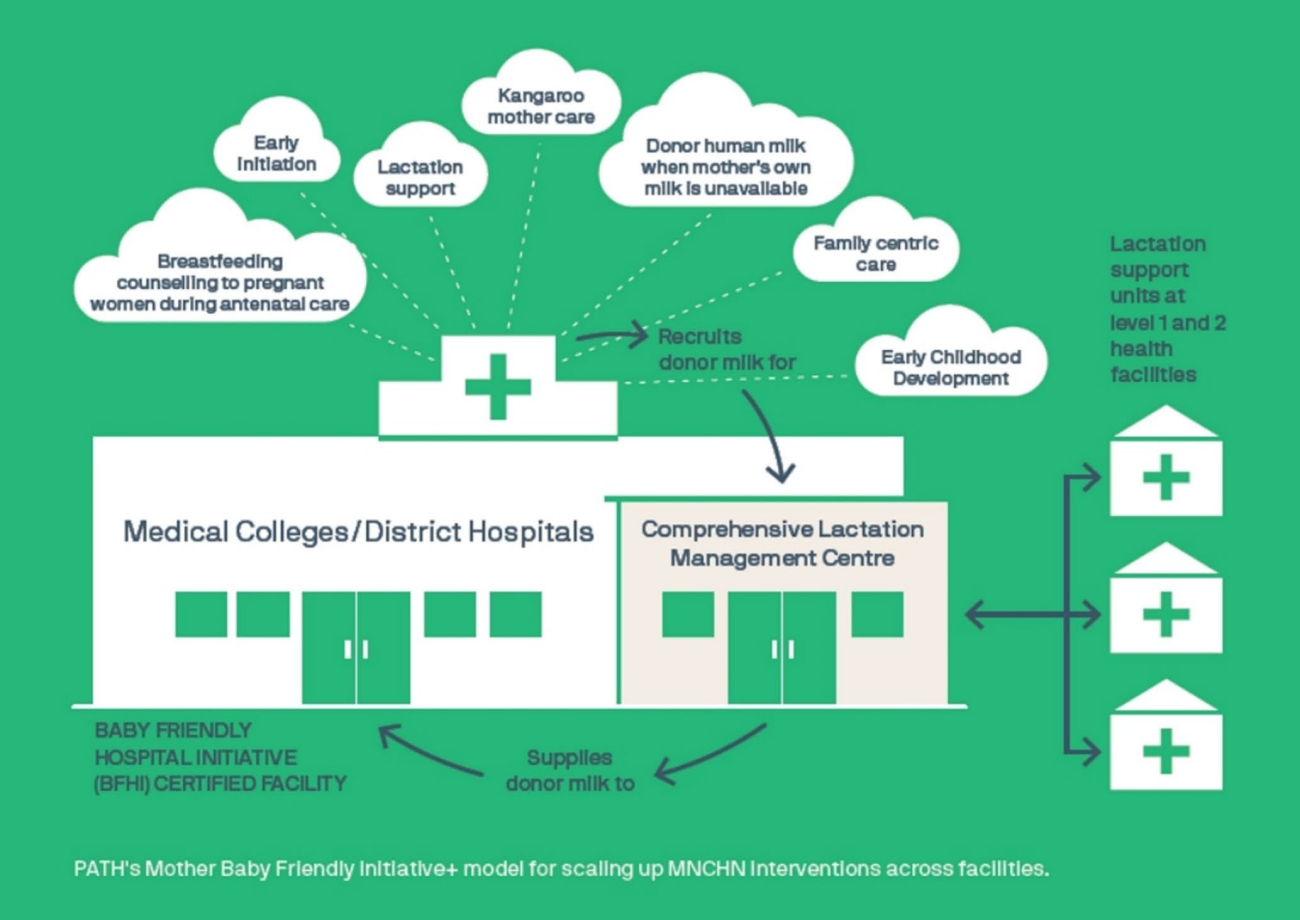
PATH’s Mother Baby Friendly Initiative+ Model for scaling up Maternal, Newborn, Child Health and Nutrition (MNCHN) Interventions.

As current evidence does not provide strong support for the use of multi-nutrient fortified breast milk in pre-term infants, more studies specially in LMICs are required for robust evidence generation (60) (Figure 6).

### 3.3 Navigating nutritional complexity in combined interventions

In low-resource settings, inadequate monitoring and evaluation mechanisms, along with limited awareness among consumers, can reduce the efficacy of fortification programs. The experience with fortification varies for different age groups and sub-groups in the population due to variations in baseline nutrient adequacy (or inadequacy), per capita consumption of different food vehicles and physiological needs (43). In India, the Food Safety and Standards Authority of India (FSSAI) followed a robust procedure to identify the optimum dosage of the fortificants that deliver only 30–50% of the Recommended Dietary Allowance (RDA). Considerations with respect to the RDA, and Tolerable Upper Limits as defined by Indian Council of Medical Research (ICMR) were also kept in mind. The analysis revealed that the current standards will only provide 30–50% of RDA given average per capita consumption levels, to fill the missing nutrient gap with no risk of overload (43).

As per global evidence interventions such as supplementation, consumption of fortified foods, and exclusive breastfeeding are safe and effective in improving nutrition outcomes among women and children (44, 45). The fortification of foods with essential vitamins and minerals is recognized as a critical priority within international development agendas (46). Correcting iron deficiency yields benefits in increased cognitive ability for children as well as greater endurance and work capacity for adults, with an estimated 8:1 cost–benefit ratio (47). These effective interventions not only combat malnutrition but also promote overall health, enhance cognitive development, and boost productivity, contributing to the nation’s socioeconomic progress. Food fortification can significantly enhance the nutritional content of additional food provided to beneficiaries under India’s Mission Poshan 2.0 (48), particularly benefiting children under 6 years old, pregnant women, and breastfeeding mothers.

## 4 Discussion

### 4.1 Importance of fortification for mother and child in the first 1,000 days

Fortification plays a pivotal role during the first 1,000 days, a critical period for ensuring the long-term health and development of both mother and child. Pregnant women have increased nutritional requirements, especially for iron and folic acid (49), and ensuring their access to fortified foods such as wheat flour and cooking oil can significantly reduce the risk of complications like anemia during pregnancy.

For infants, particularly those born prematurely or with low birth weight (LBW), breastmilk alone may not meet their nutritional needs. In such cases, breastmilk fortification with additional protein, fat, and minerals becomes necessary to support optimal growth (19). This highlights the importance of fortification efforts, which should be complemented by promoting dietary diversity. Ensuring access to a variety of nutrient-dense foods, including fruits, vegetables, legumes, and animal-source foods, is crucial to reducing malnutrition and supporting overall health.

### 4.2 Maternal nutrition and breast feeding

Maternal nutrition plays an essential role during pregnancy and lactation. Maternal malnutrition during pregnancy represents a pervasive global health concern (50). A well-nourished mother is more likely to give birth to a healthy child with fewer risks of complications such as pre-term birth or underweight infants.

In LMICs, more than 43% of children under the age of five are at risk of not reaching their full developmental potential due to the impacts of poverty and stunting (51). Proper prenatal nutrition not only supports immediate fetal health but also has long-term benefits, such as lowering the risk of chronic diseases and enhancing cognitive function and educational outcomes.

**Figure 4:**
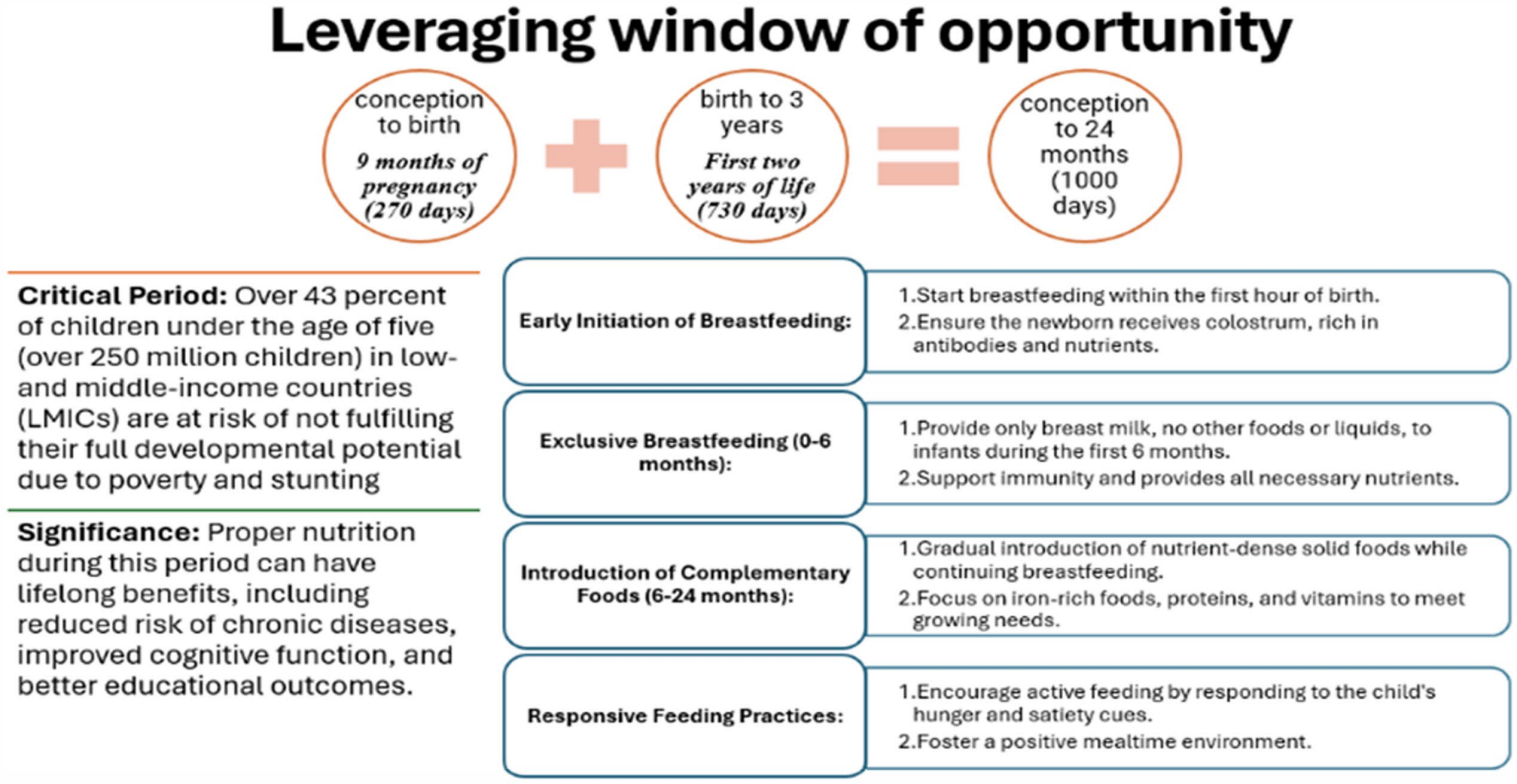
The first 1,000 days – a critical window of opportunity for child growth and development.

The early months of life are critical for establishing healthy growth patterns. Key practices include initiating breastfeeding within the first hour to provide colostrum, exclusively breastfeeding for the first 6 months to support immunity, and gradually introducing nutrient-dense complementary foods from six to 24 months (52). Responsive feeding, which involves recognizing and responding to hunger cues, fosters healthy eating habits. Interventions during this critical window can significantly improve developmental outcomes, especially in vulnerable populations, helping to mitigate stunting and other nutrition-related deficits (Figure 4).

**Figure 5:**
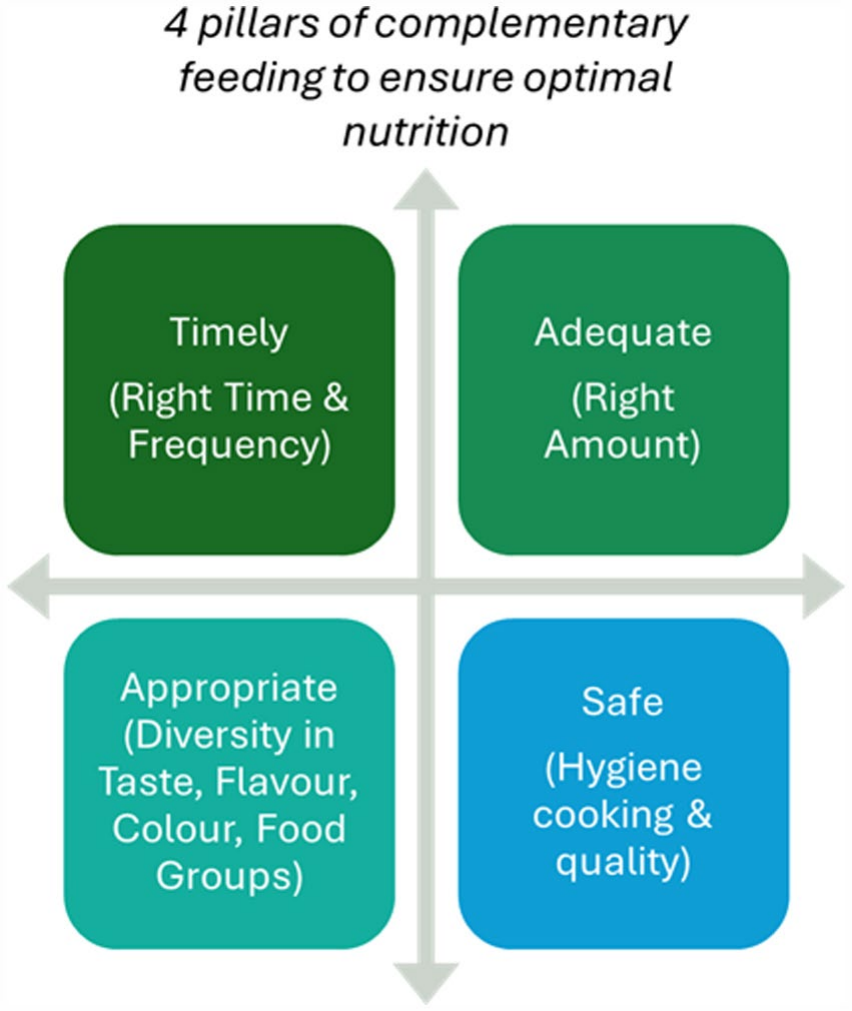
Four pillars of complementary feeding to ensure optimal nutrition.

As infants reach 6 months, their nutritional requirements outgrow what breastmilk alone can provide, necessitating the introduction of complementary foods. The four pillars of complementary feeding are timing, quality, quantity, and safety (Figure 5). However, poor-quality complementary feeding practices can lead to growth faltering, with children failing to meet developmental milestones. Ensuring nutrient-dense foods rich in iron, protein, and essential fats during this transition is critical to avoid stunting and other forms of undernutrition (53). The complementary feeding period is a sensitive time window that can influence growth, development, food preferences, and short-and long-term health outcomes.

### 4.4 Strengthening the ICDS framework and innovative approaches

The Anganwadi Services Scheme, Poshan 2.0 and Saksham Anganwadi formerly known as India’s Integrated Child Development Services (ICDS), is a comprehensive program designed to combat malnutrition and promote child health through supplemental nutrition, health services, and early childhood education. Key nutritional interventions, such as staple food fortification and breastfeeding promotion, are critical for protecting the health of mothers and young children throughout this crucial developmental period.

ICDS provides essential support to millions of children and mothers, improving their access to nutrition and healthcare, thus contributing to child development outcomes in vulnerable populations. However, changes in ICDS utilization and underweight prevalence varied considerably across states, socioeconomic, and demographic characteristics (61).

A study found that ICDS coverage was higher among younger children and the poorest households in rural areas. Multilevel logistic regression results indicated a significant association between age and the outcome variable. Severely malnourished (SAM) children living in rural areas had significantly higher odds of being covered under ICDS services (Odds Ratio 1.57; Confidence Interval: 1.35, 1.82) than their urban counterparts (62).

Pregnant and lactating mothers who received ICDS services were significant determinants of SAM coverage under the program. However, there is no evidence that ICDS is more efficient in identifying and covering SAM children compared to non-SAM children.

The ICDS program plays a pivotal role in promoting food fortification, particularly by integrating fortified foods into the supplementary nutrition provided at anganwadi centers. With its vast reach across rural and urban areas, ICDS serves as a critical distribution platform for fortified foods, especially for pregnant women, lactating mothers, and young children, who are most vulnerable to nutrient deficiencies. Fortified rice, enriched with essential vitamins and minerals, provides a reliable source of nutrition for mothers and young children, supporting their dietary needs and reducing the risk of anaemia and other deficiencies. By incorporating fortified rice into the ICDS framework, the program aims to address micronutrient deficiencies that can adversely affect health and development during this crucial window. This strategic intervention not only enhances the overall nutritional intake of beneficiaries but also aligns with the broader goals of the ICDS program to improve maternal and child health. By focusing on nutrition during the first 1,000 days, the ICDS initiative significantly contributes to long-term health outcomes and development, laying a strong foundation for the future of children in India.

### 4.5 Policy and implementation strategies

**Figure 7:**
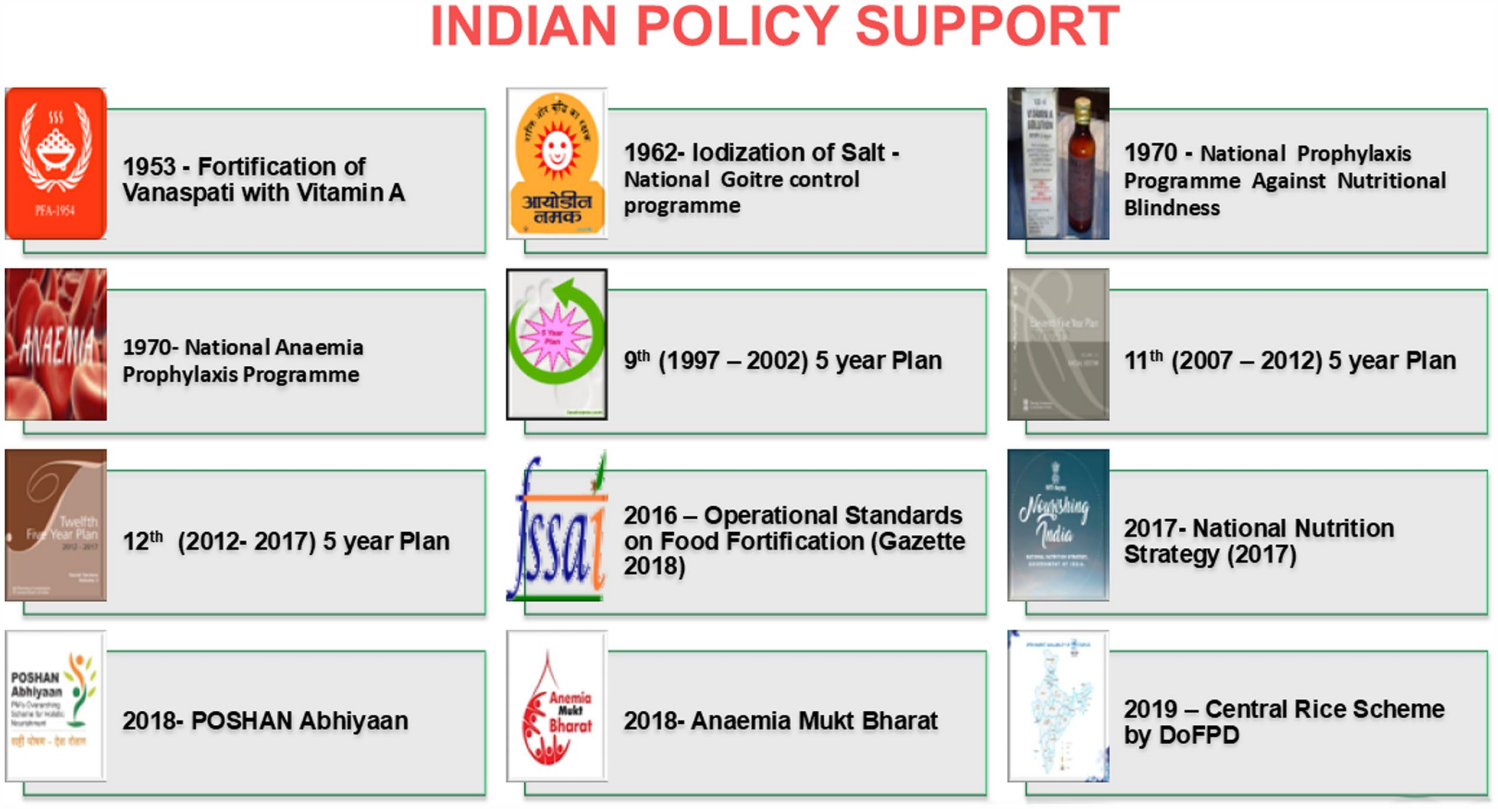
Nutrition initiatives launched by Government of India.

India has launched several nutrition initiatives (Figure 7) and launched fortification of staples such as rice, wheat flour, salt, milk and edible oil with key nutrients (40, 63). These initiatives are critical for addressing widespread micronutrient deficiencies across the population. Effective monitoring and evaluation are necessary to track the impact of fortification programs and ensure compliance with fortification standards. Digital tools and community-based monitoring can enhance the efficiency of these processes. Fortification efforts should be integrated with other public health programs, such as those addressing maternal and child health, water sanitation, and hygiene (WASH), to maximize impact. Coordinating these programs can lead to more comprehensive solutions for malnutrition.

**Figure 8:**
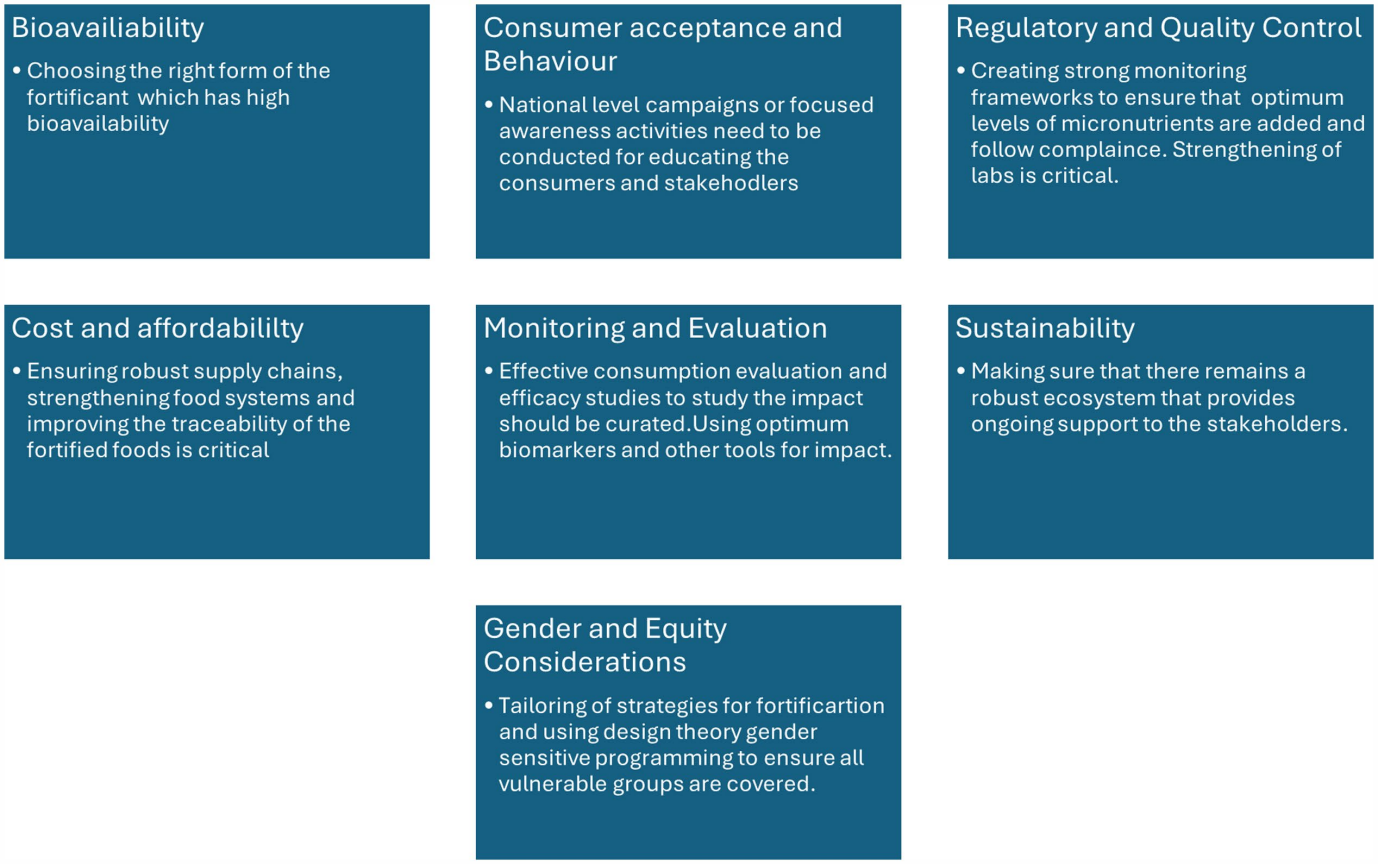
Challenges and potential solutions associated with fortification.

Public-private partnerships (PPPs) can play a crucial role in scaling up fortification efforts. By leveraging private sector expertise and resources, the ICDS can improve the reach and sustainability of nutrition interventions. Childhood stunting incurs substantial economic losses for the private sector in LMICs, amounting to billions of dollars annually in foregone sales and reduced workforce earnings. The economic rationale for investing in childhood nutrition is supported by evidence from various nutrition interventions, which highlight the dual justification for such investments—both moral and financial—pertaining to the public and private sectors. Specifically, interventions aimed at reducing stunting demonstrate returns on investment that range from US$2 to US$81 for each dollar invested per year, representing an impressive return of 100 to 8,000% across different countries (64, 65). Collaboration with local academic and research institutions can provide the ICDS program with the expertise needed to design and implement innovative nutrition interventions. Such partnerships can also support capacity building at the local levels. Food fortification has become relevant in both developing and developed countries due to the changes in dietary patterns, particularly with increase in the consumption of processed foods (66). Figure 8 presents a detailed overview of the challenges and potential solutions associated with fortification.

### 4.6 Future directions and recommendations

Advances in fortification technologies, such as biofortified crops and improved techniques that do not alter the taste or texture of food, offer great promise for addressing malnutrition. Scaling up fortification in low-resource settings, however, requires sustained political commitment, capacity building, and community engagement. The use of innovative financing mechanisms and support from international organizations will also be essential for the successful implementation of fortification programs.

Leveraging digital technology to monitor fortification efforts and track nutritional outcomes in real time can enhance the transparency and efficiency of programs like ICDS. Mobile apps and data analytics tools, for example, can play a critical role in improving program monitoring and evaluation.

Though food fortification is recognized as a highly effective approach for addressing dietary deficiencies in micronutrients like iodine, iron, and B vitamins, the success of fortification within ICDS and similar frameworks largely depends on strategic advocacy and behavior change interventions. These efforts should emphasize the importance of fortification to program beneficiaries, raising awareness around the specific nutritional needs of young children, pregnant women, and other vulnerable populations. Educational efforts should highlight the role of fortification in promoting health, covering its benefits, appropriate usage, and the practices required for different age groups.

To facilitate this awareness, comprehensive training and capacity building of Anganwadi Workers (AWWs) and other program functionaries is essential. These workers need resources, such as Information, Education, and Communication (IEC) materials and advocacy toolkits, tailored to fortification programs. Training modules should cover the nutritional importance of food fortification, detailed information on fortificants used, and specific guidelines on fortified supplementary nutrition. These resources should be accessible to both beneficiaries and program implementers, enhancing community knowledge and engagement.

Monitoring and regulatory mechanisms play a pivotal role in ensuring the quality and efficacy of fortified food distribution. Essential actions include mandatory spot checks of fortified supplementary foods, procedures for sample collection, and laboratory testing. Policymakers must also establish clear guidelines on the frequency and process for these checks. Providing spot-testing kits for qualitative assessment could be instrumental for field workers, enabling them to verify the presence of fortificants directly in communities and ensuring the integrity of the rice fortification program.

### 4.7 Recommendations for policy and practice

**Figure 9:**
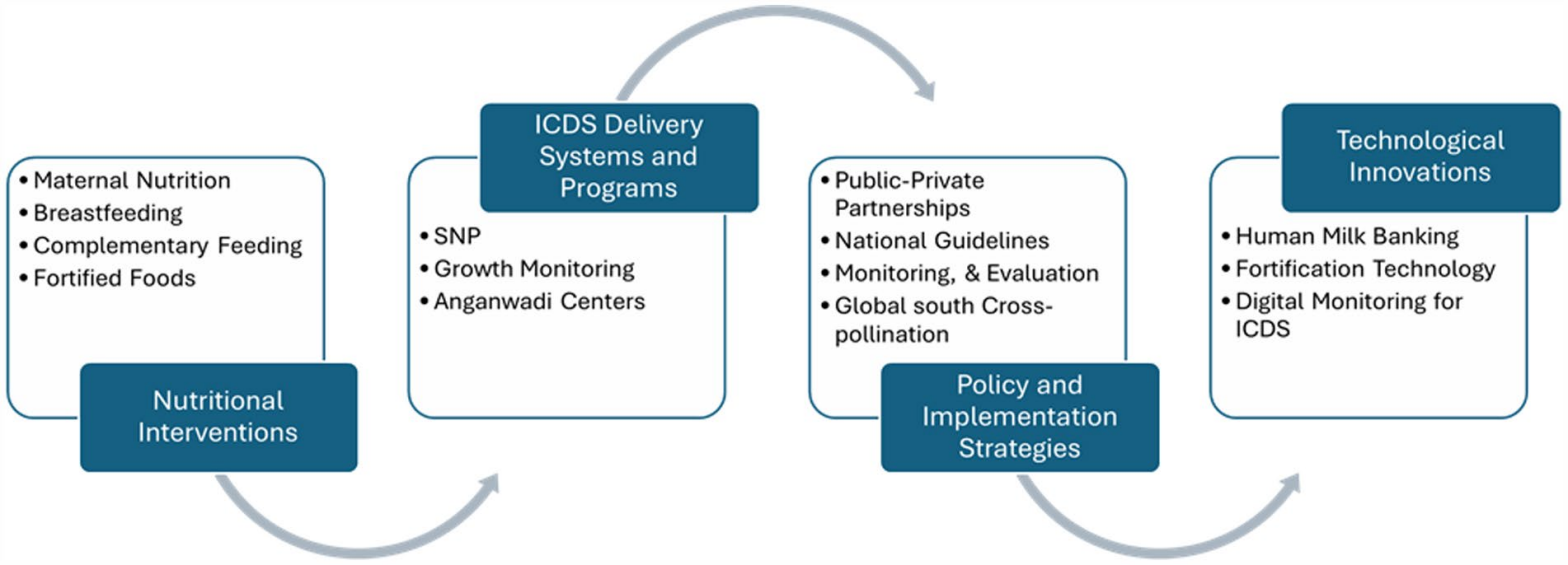
Overview of the article.

- Continued Nutritional Interventions – A sustained focus on foundational nutritional components, such as maternal nutrition, breastfeeding, and complementary feeding, remains crucial. Additionally, there is a growing recognition of the need for fortified food to address micronutrient deficiencies, alongside the continuous provision of supplementary nutrition. Such efforts are vital for ensuring both food and nutrition security. These interventions should prioritize vulnerable populations, particularly mothers and children, and be implemented consistently to address malnutrition and improve overall health outcomes. Ensuring an adequate balance in the number and mix of health workers is essential for the proper functioning of health system (67). Human resources must be carefully employed at all levels, from national to community, with a particular emphasis on guaranteeing the availability of competent health professionals, nutritionists, and program administrators. Furthermore, local community leaders and volunteers can help promote and implement these interventions. Budgets must be allocated to cover not only the direct costs of nutrition services, but also administrative expenditures such as program administration, monitoring, and evaluation, to ensure the long-term viability of the projects. While allocating resources, policymakers should pay particular attention to influencing factors. The population covered in each region and the infrastructure available in each region were identified as key and important criteria in the resource allocation process. It is suggested that these factors be considered while allocating resources to different regions of the country (68). To monitor these interventions effectively, clear indicators should be established to measure progress and impact, such as the number of health workers trained, coverage rates of nutritional services, and improvements in nutritional outcomes among target populations. Regular evaluations should be conducted at national, regional, and community levels to assess the efficiency and effectiveness of resource utilization and service delivery.
- Enhanced Delivery Systems – The integration of public-private partnerships is essential for improving the delivery of nutritional services to beneficiaries, especially mothers and children. By leveraging the strengths of both sectors, these partnerships can enhance programmatic support and extend outreach to underserved communities. This model ensures that the delivery systems are not only efficient but also sustainable, providing long-term support to the populations most in need of nutritional interventions.
- Policy and Technological Innovations – There is an urgent need to align nutrition policies with comprehensive regulatory frameworks to address the multifaceted challenges of malnutrition. Technological innovations play a pivotal role in this process, with digital tools and monitoring systems offering opportunities for improved tracking and implementation. The establishment of human milk banks, for example, represents a significant advancement in maternal and child health, ensuring that newborns receive optimal nutrition. Digitalization further facilitates transparency and accountability, enabling policymakers and practitioners to track progress and respond to emerging needs more effectively. By creating tracking systems and real time data, sustainability and long term impact can be seen. Therefore, digital technologies and AI are promising tools for health promotion and disease prevention and management, but some issues still need to be addressed, mainly those connected with patient privacy. Thus, close collaboration between healthcare institutions and research is desirable in the future (69).
- Building Capacity of Local Institutes – Strengthening the local nutrition ecosystems requires a concerted effort to build the capacity and capability of local institutions. This entails a comprehensive approach, including the provision of tailored training and educational programs for health professionals, researchers, and policymakers. Collaborating with academic institutions can further enrich the local workforce by enhancing their knowledge and skills. In addition, technical assistance and mentorship from national and international experts can facilitate the development of context-specific solutions, which are crucial for effective program implementation. Infrastructure development is another key element, with the provision of research facilities, digital tools, and access to up-to-date resources enabling local institutions to improve data collection, foster innovation, and contribute to evidence-based interventions. The sustainability of these efforts hinges on leadership training and policy advocacy, empowering local institutions to influence national nutrition policies. Moreover, building collaborative networks with non-governmental organizations (NGOs), international agencies, and the private sector will promote knowledge sharing and collective action, ultimately enhancing the capacity of local institutions to address malnutrition. A focus on sustainability is essential, ensuring that local institutions gradually assume leadership and management responsibilities, fostering long-term impact. With local support it is possible to create and sustain fieldwork for an extended period with meaningful outputs and impact. Central Sector Rice Fortification Scheme (40) demonstrates that it is possible to use healthcare professionals, students and volunteers with low-intensity training and a low-cost approach to produce action research with considerable impact and results in rapid, reliable and robust manner (70).
- Global South Cross-Pollination – Cross-pollination of ideas and best practices within the Global South is critical for advancing shared development goals, particularly in the areas of nutrition and food security. A key focus should be placed on the health of pregnant mothers and first 1,000 days of life, a crucial period for child’s growth and development, during which nutritional interventions can have a profound and lasting impact. Regional collaboration is vital for facilitating the exchange of successful strategies to address maternal and child nutrition, with particular emphasis on combating anemia and micronutrient deficiencies through the scale-up of fortified rice and other nutrition programs. The creation of knowledge-sharing platforms, as well as exchange programs for health professionals, researchers, and policymakers, will enable countries in the Global South to learn from one another’s experiences and adapt these strategies to local contexts. Establishing regional centers of excellence and organizing South–South learning events will further promote joint efforts to address shared nutritional challenges. Such initiatives will not only improve maternal and child health outcomes but also contribute to broader efforts to promote breastfeeding, human milk banking, and the expansion of fortified foods. By fostering partnerships and pooling resources, countries in the Global South can enhance their capacity to address malnutrition and food insecurity in a more effective and sustainable manner. The ongoing joint project between China and South Africa demonstrates the benefits of collaboration in developing robust remote sensing techniques for improved food security monitoring and decision making in the Global South (71).
- To address the critical health needs of LBW and preterm infants, establishing a national framework for human milk banking is essential. HMBs provide safe, pasteurized donor milk, offering life-saving benefits for infants who lack maternal milk due to medical complications. A policy for expanding HMBs should focus on integrating these banks into neonatal and public health facilities, particularly where LBW cases are high. Figure 9 provides a comprehensive summary of the article.

## 5 Conclusion

Addressing the nutritional needs of mothers and children during the first 1,000 days is key to improving long-term health outcomes and reducing malnutrition in India. Fortification programs, integrated with broader public health initiatives and supported by technological innovations, offer a promising solution for addressing micronutrient deficiencies. The success of these efforts, however, hinges on continued political commitment, effective public-private partnerships, and the engagement of local institutions. Strengthening the ICDS framework and promoting breastfeeding and human milk banking are critical steps toward ensuring that every child receives the nutrients they need for a healthy start in life.

## References

[1] Black RE Allen LH Bhutta ZA Caulfield LE De Onis M Ezzati M . Maternal and child undernutrition: global and regional exposures and health consequences. Lancet Lond Engl. (2008) 371:243–60. doi: 10.1016/S0140-6736(07)61690-0, PMID: 18207566

[2] United Nations. *The sustainable development goals report*. (2024). Available at: https://unstats.un.org/sdgs/report/2024/The-Sustainable-Development-Goals-Report-2024.pdf.

[3] FAO, IFAD, UNICEF, WFP, WHO. *The state of food security and nutrition in the world 2024*. FAO, IFAD; UNICEF; WFP; WHO; (2024). Available at: https://openknowledge.fao.org/handle/20.500.14283/cd1254en.

[4] Csölle I Felső R Szabó É Metzendorf MI Schwingshackl L Ferenci T . Health outcomes associated with micronutrient-fortified complementary foods in infants and young children aged 6–23 months: a systematic review and meta-analysis. Lancet Child Adolesc Health. (2022) 6:533–44. doi: 10.1016/S2352-4642(22)00147-X, PMID: 35753314 PMC9279162

[5] Das JK Salam RA Kumar R Bhutta ZA. Micronutrient fortification of food and its impact on woman and child health: a systematic review. Syst Rev. (2013) 2:67. doi: 10.1186/2046-4053-2-67, PMID: 23971426 PMC3765883

[6] Scott JA. The first 1000 days: a critical period of nutritional opportunity and vulnerability. Nutr Diet. (2020) 77:295–7. doi: 10.1111/1747-0080.12617, PMID: 32478460

[7] World Health Organization. *United Nations Children’s fund, World Health Organization, World Bank. Levels and trends in child malnutrition: Key findings of the 2019 edition*. (2019).

[8] Victora CG Christian P Vidaletti LP Gatica-Domínguez G Menon P Black RE. Revisiting maternal and child undernutrition in low-income and middle-income countries: variable progress towards an unfinished agenda. Lancet. (2021) 397:1388–99. doi: 10.1016/S0140-6736(21)00394-9, PMID: 33691094 PMC7613170

[9] Beluska-Turkan K Korczak R Hartell B Moskal K Maukonen J Alexander DE . Nutritional gaps and supplementation in the first 1000 days. Nutrients. (2019) 11:2891. doi: 10.3390/nu11122891, PMID: 31783636 PMC6949907

[10] Islam MS Zafar Ullah AN Mainali S Imam MA Hasan MI. Determinants of stunting during the first 1, 000 days of life in Bangladesh: a review. Food Sci Nutr. (2020) 8:4685–95. doi: 10.1002/fsn3.1795, PMID: 32994930 PMC7500796

[11] Bordeleau M Fernández de Cossío L Chakravarty MM Tremblay MÈ. From maternal diet to neurodevelopmental disorders: a story of neuroinflammation. Front Cell Neurosci. (2021) 14:612705. doi: 10.3389/fncel.2020.612705, PMID: 33536875 PMC7849357

[12] Dandona R Kumar GA Henry NJ Joshua V Ramji S Gupta SS . Subnational mapping of under-5 and neonatal mortality trends in India: the global burden of disease study 2000–17. Lancet. (2020) 395:1640–58. doi: 10.1016/S0140-6736(20)30471-2, PMID: 32413293 PMC7262604

[13] Chattopadhyay N Aneja S. The status of early childhood development in India: will we reach the countdown to 2030 targets? Indian Pediatr. (2021) 58:4–10. doi: 10.1007/s13312-021-2348-6 34687181

[14] ICF. *International Institute for Population Sciences (IIPS), ICF. National Family Health Survey (NFHS-5) 2019–21*. (2021). Available at: https://dhsprogram.com/pubs/pdf/FR375/FR375.pdf.

[15] ICF. *International institute for population sciences (IIPS). National Family Health Survey (NFHS-4) 2015–16*. (2017). Available at: https://dhsprogram.com/pubs/pdf/fr.339/fr339.pdf.

[16] Abbaspour N Hurrell R Kelishadi R. Review on iron and its importance for human health. J Res Med Sci. (2014) 19:164–74. PMID: 24778671 PMC3999603

[17] Dobe M Garg P Bhalla G. Fortification as an effective strategy to bridge iron gaps during complementary feeding. Clin Epidemiol Glob Health. (2018) 6:168–71. doi: 10.1016/j.cegh.2017.11.001

[18] World Health Organization. *Multiple-micronutrient supplementation for women during pregnancy*. Available at: https://www.who.int/tools/elena/review-summaries/micronutrients-pregnancy--multiple-micronutrient-supplementation-for-women-during-pregnancy.

[19] Arslanoglu S Boquien CY King C Lamireau D Tonetto P Barnett D . Fortification of human Milk for preterm infants: update and recommendations of the European Milk Bank Association (EMBA) working group on human Milk fortification. Front Pediatr. (2019) 7:76. doi: 10.3389/fped.2019.00076 30968003 PMC6439523

[20] Arslanoglu S Moro GE Tonetto P De Nisi G Ambruzzi AM Biasini A . Recommendations for the establishment and operation of a donor human milk bank. Nutr Rev. (2023) 81:1–28. doi: 10.1093/nutrit/nuad012, PMID: 36892193 PMC9997086

[21] Osendarp SJM Martinez H Garrett GS Neufeld LM De-Regil LM Vossenaar M . Large-scale food fortification and biofortification in low-and middle-income countries: a review of programs, trends, challenges, and evidence gaps. Food Nutr Bull. (2018) 39:315–31. doi: 10.1177/0379572118774229, PMID: 29793357 PMC7473077

[22] Miller GD Kanter M Rycken L Comerford KB Gardner NM Brown KA. Food systems transformation for child health and well-being: the essential role of dairy. Int J Environ Res Public Health. (2021) 18:10535. doi: 10.3390/ijerph181910535, PMID: 34639835 PMC8507772

[23] Olson R Gavin-Smith B Ferraboschi C Kraemer K. Food fortification: the advantages, disadvantages and lessons from sight and life programs. Nutrients. (2021) 13:1118. doi: 10.3390/nu13041118, PMID: 33805305 PMC8066912

[24] Martorell R Ascencio M Tacsan L Alfaro T Young MF Addo OY . Effectiveness evaluation of the food fortification program of Costa Rica: impact on anemia prevalence and hemoglobin concentrations in women and children. Am J Clin Nutr. (2015) 101:210–7. doi: 10.3945/ajcn.114.097709, PMID: 25527765 PMC5884061

[25] Hemalatha R Reddy NS Challa S Venkatesh K Pullakhandam R Nandeep ER . *Efficacy and safety of iron fortified rice in India - a white paper*. Indian Council for Medical Research (2023). Available at: https://www.nin.res.in/downloads/whitepaper.pdf.

[26] Kachhawa K Mogra R. Large-scale fortification of Rice in India and its distribution in public distribution system. Indian J Public Adm. (2023) 69:779–87. doi: 10.1177/00195561231196214, PMID: 39866753

[27] Thakur S Singh A Insa B Sharma S. Food fortification in India as malnutrition concern: a global approach. Sustain Food Technol. (2023) 1:681–95. doi: 10.1039/D3FB00079F

[28] Fatemi SF Irankhah K Kruger J Bruins MJ Sobhani SR. Implementing micronutrient fortification programs as a potential practical contribution to achieving sustainable diets. Nutr Bull. (2023) 48:411–24. doi: 10.1111/nbu.12630, PMID: 37503811

[29] Keats EC Neufeld LM Garrett GS Mbuya MNN Bhutta ZA. Improved micronutrient status and health outcomes in low-and middle-income countries following large-scale fortification: evidence from a systematic review and meta-analysis. Am J Clin Nutr. (2019) 109:1696–708. doi: 10.1093/ajcn/nqz023, PMID: 30997493 PMC6537942

[30] World Health Organization. Guideline: Fortification of rice with vitamins and minerals as a public health strategy. Geneva: World Health Organization (2018). 30307723

[31] Chowdhury S Ray D. Micronutrient deficiency in Indian diet. Int J Sci Res Eng Manag. (2024) 8:1–5. doi: 10.55041/IJSREM33586, PMID: 39844068

[32] Van Wonderen D Melse-Boonstra A Gerdessen JC. Iron bioavailability should be considered when modeling omnivorous, vegetarian, and vegan diets. J Nutr. (2023) 153:2125–32. doi: 10.1016/j.tjnut.2023.05.011, PMID: 37182693

[33] Chungchunlam SMS Moughan PJ. Comparative bioavailability of vitamins in human foods sourced from animals and plants. Crit Rev Food Sci Nutr. (2024) 64:11590–625. doi: 10.1080/10408398.2023.2241541, PMID: 37522617

[34] FSSAI. *About us: Fortification*. Available at: https://fortification.fssai.gov.in/aboutus.

[35] Neufingerl N Eilander A. Nutrient intake and status in children and adolescents consuming plant-based diets compared to meat-eaters: a systematic review. Nutrients. (2023) 15:4341. doi: 10.3390/nu15204341, PMID: 37892416 PMC10609337

[36] Grosse SD Waitzman NJ Romano PS Mulinare J. Reevaluating the benefits of folic acid fortification in the United States: economic analysis, regulation, and public health. Am J Public Health. (2005) 95:1917–22. doi: 10.2105/AJPH.2004.058859, PMID: 16195513 PMC1449459

[37] Duvenage SS Schönfeldt HC. Impact of South African fortification legislation on product formulation for low-income households. J Food Compos Anal. (2007) 20:688–95. doi: 10.1016/j.jfca.2007.04.001

[38] Galani YJH Orfila C Gong YY. A review of micronutrient deficiencies and analysis of maize contribution to nutrient requirements of women and children in eastern and southern Africa. Crit Rev Food Sci Nutr. (2022) 62:1568–91. doi: 10.1080/10408398.2020.1844636, PMID: 33176441

[39] Dasher R Kapoor A Anand M. *Rice fortification in India: Progression and insights*. Institute for Competitiveness, Stanford US Asia Technology Management Centre (2024). Available at: https://www.competitiveness.in/wp-content/uploads/2023/10/Report_Rice_Fortifcation_Web_version.pdf.

[40] Press Information Bureau. *Ministry of Consumer Affairs, food & public distribution*. (2024). Available at: http://pib.gov.in/PressNoteDetails.aspx?NoteId=153272.

[41] Mahapatra S Parker ME Dave N Zobrist SC Shajie Arul D King A . Micronutrient-fortified rice improves haemoglobin, anaemia prevalence and cognitive performance among schoolchildren in Gujarat, India: a case-control study. Int J Food Sci Nutr. (2021) 72:690–703. doi: 10.1080/09637486.2020.1855126, PMID: 33427528

[42] World Health Organization, Food and Agricultural Organization, of the United Nations. Guidelines on food fortification with micronutrients. In: Guidelines on food fortification with micronutrients. Geneva: World Health Organization (2006).

[43] Duggal M Sesikeran B Arlappa N Nair S Shekhar V Sabharwal V. Large-scale staple food fortification as a complementary strategy to address vitamin and mineral vulnerabilities in India: a critical review. Indian J Public Health. (2022) 66:313–20. doi: 10.4103/ijph.ijph_708_22, PMID: 36149111

[44] Bhutta ZA Das JK Rizvi A Gaffey MF Walker N Horton S . Evidence-based interventions for improvement of maternal and child nutrition: what can be done and at what cost? Lancet. (2013) 382:452–77. doi: 10.1016/S0140-6736(13)60996-4, PMID: 23746776

[45] Bhutta ZA. Early nutrition and adult outcomes: pieces of the puzzle. Lancet. (2013) 382:486–7. doi: 10.1016/S0140-6736(13)60716-3, PMID: 23541369

[46] Muthayya S Hall J Bagriansky J Sugimoto J Gundry D Matthias D . Rice fortification: an emerging opportunity to contribute to the elimination of vitamin and mineral deficiency worldwide. Food Nutr Bull. (2012) 33:296–307. doi: 10.1177/156482651203300410, PMID: 23424896

[47] Jamison DT Breman JG Measham AR Alleyne G Claeson M Evans DB . *Priorities in health*. Washington, DC: The International Bank for Reconstruction and Development/The World Bank (2006). 21089239

[48] Ministry of Women and Child Development. MISSION POSHAN 2.0. (2023). Available at: https://pib.gov.in/Pressreleaseshare.aspx?PRID=1910097.

[49] Lactation I of M (US) C on NSDP. *Iron nutrition during pregnancy. In: Nutrition during Pregnancy: Part I Weight Gain: Part II Nutrient Supplements*. National Academies Press, US (1990). 25144018

[50] De Reis AEM Teixeira IS Maia JM Luciano LAA Brandião LM Silva MLS . Maternal nutrition and its effects on fetal neurodevelopment. Nutrition. (2024) 125:112483. doi: 10.1016/j.nut.2024.112483, PMID: 38823254

[51] Gil JD Ewerling F Ferreira LZ Barros AJ. Early childhood suspected developmental delay in 63 low-and middle-income countries: large within- and between-country inequalities documented using national health surveys. J Glob Health. (2020) 10:010427. doi: 10.7189/jogh.10.010427, PMID: 32566165 PMC7295453

[52] World Health Organization. *Infant and young child feeding*. Available at: https://www.who.int/news-room/fact-sheets/detail/infant-and-young-child-feeding.

[53] D’Auria E Borsani B Pendezza E Bosetti A Paradiso L Zuccotti GV . Complementary feeding: pitfalls for health outcomes. Int J Environ Res Public Health. (2020) 17:7931. doi: 10.3390/ijerph17217931, PMID: 33137971 PMC7662522

[54] FFOODS Spectrum. (2023). *Understanding barriers and facilitators for rice fortification in India*. Available at: https://nuffoodsspeunderstandingctrum.in/2023/04/05/-barriers-and-facilitators-for-rice-fortification-in-india.html.

[55] Chugh Sachdeva R Mondkar J Shanbhag S Manuhar M Khan A Dasgupta R . A qualitative analysis of the barriers and facilitators for breastfeeding and kangaroo mother care among service providers, mothers and influencers of neonates admitted in two urban hospitals in India. Breastfeed Med. (2019) 14:108–14. doi: 10.1089/bfm.2018.0177, PMID: 30676061

[56] Moro GE Girard M Peila C Garcia N Escuder-Vieco D Keller K . New alternatives to holder pasteurization in processing donor milk in human milk banks. Front Nutr. (2024) 11:381. doi: 10.3389/fnut.2024.1409381 PMC11234892 38988859

[57] Sachdeva RC Mondkar J Shanbhag S Sinha MM Khan A Dasgupta R. A landscape analysis of human Milk banks in India. Indian Pediatr. (2019) 56:663–8. doi: 10.1007/s13312-019-1590-7, PMID: 31477647

[58] Bergner EM Taylor SN Gollins LA Hair AB. Human Milk fortification: a practical analysis of current evidence. Clin Perinatol. (2022) 49:447–60. doi: 10.1016/j.clp.2022.02.010, PMID: 35659096

[59] Salas AA Gunawan E Nguyen K Reeves A Argent V Finck A . Early human Milk fortification in infants born extremely preterm: a randomized trial. Pediatrics. (2023) 152:e2023061603. doi: 10.1542/peds.2023-061603, PMID: 37551512 PMC10471508

[60] World Health Organization. *Multi-nutrient fortification of human milk for preterm infants*. Available at: https://www.who.int/tools/elena/review-summaries/feeding-vlbw-infants--multi-nutrient-fortification-of-human-milk-for-preterm-infants.

[61] Singh SK Chauhan A Alderman H Avula R Dwivedi LK Kapoor R . Utilization of integrated child development services (ICDS) and its linkages with undernutrition in India. Matern Child Nutr. (2024) 20:e13644. doi: 10.1111/mcn.13644, PMID: 38586943 PMC11168363

[62] Chakraborty R Joe W Shankar Mishra U Rajpal S. Integrated child development service (ICDS) coverage among severe acute malnourished (SAM) children in India: a multilevel analysis based on national family health survey-5. PLoS One. (2024) 19:e0294706. doi: 10.1371/journal.pone.0294706, PMID: 38330040 PMC10852256

[63] Press Information Bureau. *Centre to supply fortified rice throughout the country by 2024 with the help of central schemes like targeted public distribution system (TPDS) and other welfare schemes (OWS)*. (2022). Available at: https://pib.gov.in/pib.gov.in/Pressreleaseshare.aspx?PRID=1845414.

[64] European Society for Pediatric. World Health Organization (WHO) guideline on the complementary feeding of infants and young children aged 6−23 months 2023: a multisociety response. J Pediatr Gastroenterol Nutr. (2024) 79:181–8. doi: 10.1002/jpn3.12248 38743631

[65] Galasso E Wagstaff A. The aggregate income losses from childhood stunting and the returns to a nutrition intervention aimed at reducing stunting. Econ Hum Biol. (2019) 34:225–38. doi: 10.1016/j.ehb.2019.01.010, PMID: 31003858

[66] Ofori KF Antoniello S English MM Aryee ANA. Improving nutrition through biofortification–a systematic review. Front Nutr. (2022) 9:655. doi: 10.3389/fnut.2022.1043655 PMC9784929 36570169

[67] Witter S Hamza MM Alazemi N Alluhidan M Alghaith T Herbst CH. Human resources for health interventions in high- and middle-income countries: findings of an evidence review. Hum Resour Health. (2020) 18:43. doi: 10.1186/s12960-020-00484-w, PMID: 32513184 PMC7281920

[68] Nouri S Riahi L Nabi KH Jahangiri K. Identifying key factors related to the resource allocation in the health sector of the Iranian oil industry: application of DEMATEL method. Health Scope. (2020) 9:728. doi: 10.5812/jhealthscope.97728, PMID: 39416803

[69] Salinari A Machì M Armas Diaz Y Cianciosi D Qi Z Yang B . The application of digital technologies and artificial intelligence in healthcare: an overview on nutrition assessment. Diseases. (2023) 11:97. doi: 10.3390/diseases11030097, PMID: 37489449 PMC10366918

[70] Buckner L Carter H Ahankari A Banerjee R Bhar S Bhat S . Three-year review of a capacity building pilot for a sustainable regional network on food, nutrition and health systems education in India. BMJ Nutr Prev Health. (2021) 4:59–68. doi: 10.1136/bmjnph-2020-000180 PMC8258077 34308113

[71] Balz T Gao R Musakwa W Magidi J Shao Z. South-to-south cooperation in multi-source satellite data for improving food security. Int arch Photogramm remote Sens spat. Inf Sci. (2024) XLVIII-1-2024:19–24. doi: 10.5194/isprs-archives-XLVIII-1-2024-19-2024, PMID: 38859159

